# On the consequences of firm growth

**DOI:** 10.1177/02662426221074053

**Published:** 2022-09-17

**Authors:** Mark Freel, Ian Gordon

**Affiliations:** Telfer School of Management, 6363 University of Ottawa, Ottawa, ON, Canada;; Lancaster University Management School, Lancaster, UK; Business School, University of Edinburgh, Edinburgh, UK

**Keywords:** growth, case studies, motivation, ambidexterity

## Abstract

Recent contributions to the literature on small firm growth have been marked by a growing sense of frustration with the state-of-the-art and what it implicates in both theory and policy. In short, while growth episodes appear relatively common, a tiny proportion of firms sustain growth and ‘scale’. This calls into question the very basis upon which policies seeking to target high growth firms (HGFs) rest. In addition, it cautions against perspectives that view growth as the essence of entrepreneurship. In this paper, we argue that understanding the frequency of growth episodes and the rarity of sustained growth requires a better understanding of growth consequences. To this end, we describe case study evidence from ambitious entrepreneurs whose firms experienced an episode of high growth followed by longer periods of mixed performance. Our goal is to shed light on how the experience of growing affects further growth. Our data provide initial insights into the mechanisms linking past growth to growth motivations and into the ways in which past growth lays the foundations for future performance.

## Introduction

Our interest in the consequences of growth in smaller firms was initially occasioned by rereading Nicholls-Nixon and Charlene (2005). In a field where consensus is sufficiently rare as to be remarkable, the notion that a small number of firms are responsible for a larger part of the economic gains has acquired the status of ‘stylised fact’. While Nicholls-Nixon’s (2005, p. 77) characterisation of rapid growth ‘as the business equivalent of a birdie, a touchdown, or a home run on the field of dreams’ might appear consistent with this orthodoxy, it was her observations on the episodic nature of high growth that led us to reflect on growth consequences. Evidence on the skewed distribution of economic returns is longstanding and widespread (Coad et al., 2014a). However, the typical approach to identifying high growth firms is in the cross-section; recognising, categorising, measuring, studying, and so on, these firms at some point in time. Nicholls-Nixon’s (2005, p.78) finding that ‘in the 22 years since *Inc. Magazine* began ranking high-growth companies, only 69 have made it onto the list two or more times implies at least two things. First, that high growth is astonishingly difficult to replicate; and second, that many more firms experience an episode of high growth than observations at a specific point in time approaches might suggest.

The first is reinforced by recent evidence on growth persistence. Simply put, past growth does not seem to be a useful predictor of future growth in samples of small and young firms. Indeed, high growth firms are as likely to be found among the previous period’s worst performers as they are among the best performers (Coad, et al., 2015; Daunfeldt and Halvarsson, 2015; Henrekson and Johansson, 2010). In short, most growth firms are ‘one hit wonders’ (Daunfeldt and Halvarsson, 2015). The second issue is nicely illustrated by Derbyshire and Garnsey (2015). Responding to suggestions that ‘firm growth is well-approximated by a random walk’ (Coad et al., 2013, p. 615), these authors record the four year growth, stability and decline of almost 40,000 firms started in the UK in 2005. Persistent growth (i.e. growth in all four years) is, indeed, very rare – only three firms grew in all years. However, perhaps more interestingly, 12,297 firms (31% of the sample) were recorded as growing in at least one of those years.

Accepting that many firms grow, but that very few enjoy sustained periods of growth, ought to have us thinking about the consequences of growth at least as much as the causes (Achtenhagen et al., 2010). Of course, failure to continue to grow may reflect external factors such as new competition, changing regulations, slumping demand, technology shocks and so on. However, the extent of the evidence on the episodic nature of firm growth suggests that explanations that rely upon external influences alone are unlikely to be particularly useful. Rather, it seems that something happens to the entrepreneur and within the firm, as they grow, that limits the likelihood of growing again. Rather than success breeding success, it is just as likely to breed failure (Delmar et al., 2013; Zhou and Van der Zwan, 2019) and more likely to lead to neither future success or failure.

This, of course, is not a new idea. The organisational lifecycle models of firm growth that enjoyed considerable prominence in the early entrepreneurship literature often explicitly recognised the organisational challenges that accompany growth (see Levie and Lichtenstein (2010) for a review). In one of the more popular examples, Greiner (1989) anticipated that a firm’s initial growth will slow as inefficiencies become apparent with increasing scale and ‘founders find themselves burdened by unwanted managerial responsibilities’ (p. 6). In a similar vein, Hambrick and Crozier (1985) identified underdeveloped systems and limited managerial acumen as characteristics of high growth firms that ‘stumble’. However, in these accounts, not continuing to grow results from managerial missteps or a failure to rise to new challenges. Not growing is aberrant. Unfortunately, this normative perspective on growth is likely to bias research designs and lead to restricted theorising (Dutta and Thornhill, 2008), leaving many of us ‘dazed and confused by the wild hype’ surrounding high growth firms (Aldrich and Ruef, 2018, p. 458).

This, then, was our research question: Why do apparently good firms fail to sustain growth? Or, to ask the question differently, what happens to those firms or to the people running them that results in high growth occurring only once? In this way, our concern was primarily with the consequences of growth and not with its causes, and with how these consequences bear on the likelihood of growing, or not growing, again^1^. A focus on consequences is consistent with a view of growth as an intermediate outcome (Achtenhagen et al., 2010), and better recognises the multiplicity of goals that entrepreneurs pursue.

Having reached a determination that the study of growth consequences was likely to be revealing, our initial thought was to rush to the sophisticated large-scale datasets increasingly available to researchers. Unfortunately, when we got to the data^2^, we struggled to articulate good questions. The focus on growth as an outcome has led the growth literature to be concerned with causes and constraints. Not with consequences. This is compounded by a sense that ‘individual cognitive decision processes or micro-foundations has been a particularly problematic omission in the literature on the growth of entrepreneurial ventures’ (Wright and Stigliani, 2013, p. 4). While the existing growth literature, and especially the early work on organisational life-cycles, offered us some guidance, our intuition was that the specification of ‘good’ hypotheses required richer data collected specifically for that purpose. What we wanted was a smaller number of case studies that represented good firms – those that had enjoyed high growth in the past and had articulated a desire to grow further – that had been unable to sustain growth in line with the early ambitions of the founders. With the richer data that these case studies would provide, we hoped to begin to develop preliminary models that, in turn, could be articulated as hypotheses that were testable with the survey and administrative data that we had access to. This is in line with calls for ‘the increased use of data collection methods that focus on what entrepreneurs actually do’ (Mueller et al., 2012), leveraging mixed methods approaches to the study of complex entrepreneurial phenomena that may allow researchers to generate additional insights (Maula and Stam, 2020).

We suspect that this is a rather unusual article, at least insofar as it reports only part of a research project. The research detailed here draws on archival and interview data from six case companies. In doing this, our goal is twofold: First, to begin to uncover the consequences of growth that bear most directly on the likelihood of (not) sustaining growth. And, second, to elaborate a general case for the further study of growth consequences. In the next section, we introduce our cases, outline their selection and our processes of data collection and analysis. In the following sections, we explore patterns in the case study data that appear to inform our research question. We conclude with suggestions for future research and with reflections on the potential implications for policy and practice of a better understanding of growth consequences.

## Finding ‘good’ firms that don’t grow

What is a good firm? Of course, there are likely to be many reasonable answers to that question. For our current purposes, we consider a ‘good firm’ to be one that had enjoyed at least one period of high growth and that continued to trade profitably for several years after the growth episode. These are not the MUPPETS (Marginal Undersized Poor Performance Enterprises) provocatively identified by Nightingale and Coad (2014). But neither are they – at least any longer – the gazelles or unicorns that dominate the entrepreneurship menagerie and distract from our understanding of everyday entrepreneurship (Aldrich and Ruef, 2018).

Specifically, our purposeful sampling (Neergaard, 2007) sought previously growth-oriented firms that had enjoyed mixed fortunes since their initial high growth episode but who, nonetheless, were profitable enterprises. We also required access to historical performance data and to some means of identifying prior goals and aspirations beyond simple retrospective reporting, given longstanding concerns over recall bias in research on entrepreneurial motivations (Cassar, 2007). This, clearly, was a tall order. Fortunately, through our professional networks, we were aware of a group of firms who might meet our criteria. These firms had participated in a growth-oriented leadership development programme offered by a university in the north of England in 2010 and 2011. Participants in the programme had been part of a prior development programme, had previously enjoyed a period of high growth and had signalled, through their involvement in this subsequent programme, ambition for further business growth and development. Our sampling strategy was clearly both opportunistic and purposive.

**Figure 1.**
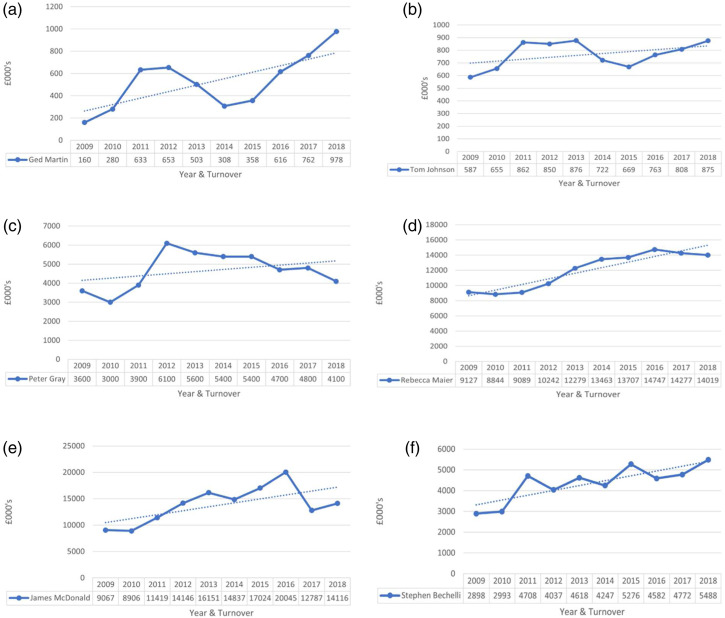
Recent firm sales performance.

Nonetheless, in selecting our sample, we were conscious of longstanding debates in the literature regarding suitable growth metrics (Delmar, 2006; Brännback et al., 2014). Employment growth and sales growth dominate empirical studies, but are conceptually distinct and demonstrate low concurrent validity (Shepherd and Wiklund, 2009). Moreover, while employment growth may be attractive to policymakers hoping for job creation (Shane, 2009), it is not clear that entrepreneurs value increased employment quite so much. In much the same way, while sales figures are attractive to researchers in search of relatively standardised and comparable data, growth has been shown to be a multidimensional phenomenon for practitioners, strongly emphasising internal development (Achtenhagen et al., 2010). The participation of our case firms in university-led development programmes, and their continued engagement as entrepreneurs in residence and guest lecturers, provided us with a variety of data that assured us that our cases represented firms that had enjoyed prior high growth episodes (in employment and sales) and undergone significant internal development that would allow the entrepreneurs to articulate experiences of growing. Revealingly, although sales and employment were rarely stated goals, our entrepreneurs consistently used sales levels to punctuate their stories of growth and development; as targets set, losses encountered, or milestones reached. This is reflected in our use of sales to sketch the more recent variable performance of our case firms (Figure 1). However, it does not diminish the essential multidimensionality of growth or the multiplicity of entrepreneurial goals. We return to these issues in our discussion.

We also use the terms ‘rapid growth’ and ‘high growth’ colloquially and interchangeably. While the former is explicitly concerned with time, the latter is more generally concerned with extent. However, in practice, a temporal dimension is typical in studies of high growth (Coad et al., 2014a), with windows of a few years commonly adopted in an attempt to smooth the data. Indeed, this approach has been formalised in the OECD and Eurostat (Eurostat, 2007) definition of High Growth Firms (capitalised), as firms with at least 10 employees in the base year and annualised employment growth exceeding 20% during a three year period. While this approach has proved to be popular in policy circles, it ignores the reality that most high growth firms experience their growth event in a single year (Daunfeldt and Halvarsson, 2015; Hölzl, 2014). Perhaps accordingly, empirical research continues to be marked by different approaches to measuring high growth, but by the common characterisation of high growth firms as firm who grow rapidly (Demir et al., 2017).

Finally, our cases are highly varied. Three had their origins in family businesses that predated the involvement of our entrepreneurs, while three were de novo ventures. Two of the companies are engaged in manufacturing, three are in Business-to-Business (B2B) services, and one is in retail. At the time we interviewed the entrepreneurs, venture sales ranged from £875,000 to over £14,000,000. Employment ranged from 18 to 99. However, our entrepreneurs had in common an experience of high growth and a prior commitment to further growth and development. Some small effort has been made to disguise the companies^3^, but the essence of each is set out in the vignettes below.

Case A Ged Martin and Safety First

Safety First was started by Ged Martin in late 2002. The business delivers electrical safety, fire safety and health and safety assessments and services to businesses throughout the UK. Having resigned from his previous employment, disillusioned with labour practices in a large, unionised environment, Ged set up on his own, providing safety training ‘until I worked out what was next…[because]…bills still needed paying’^4^. The ‘business had trundled along for six or seven years, doing next to nothing with regards to turnover, and very little on the profit side’. However, sales almost doubled between 2009 and 2010 and doubled again in 2011. The growth was largely driven by client acquisition; especially the acquisition of two major clients within a three week period in 2010. As the data in Figure 1 show, this was followed by a rapid decline in performance before a recovery in 2016. At the time we interviewed Ged, the firm directly employed 18 people, retaining the services of a further 45 freelance consultants, and had recorded sales of around £1m.

Case B Tom Johnson and Food Services

After a health scare forced Tom Johnson to retire from the police force in 1990, he joined the family food services business. The business entered insolvency in 2007. This was Tom’s entrepreneurial moment, acquiring assets from the administrator and, ‘just with the bits that I wanted’, starting over again. The new business consisted of a production facility and three retail sites. The first couple of years of the business were concerned with settling debts and rebuilding reputation ‘because one of the things you learn when you go into liquidation is no one wants to trade with you’. Growth came in 2009–10 with a shift from fixed to mobile retail. As Tom explained, ‘retail is for me, but not through a shop. Because a shop’s got leases and rents and rates and it’s fixed and I can’t move it’. Sales reach £862,000 in 2011, up 32% from the previous year and more than double 2008 sales. Employment peaked at 37 employees. As Figure 1 shows, this was followed by two years of stability and then two years of decline. At the time we interviewed Tom in 2019, sales had gradually recovered to 2011 levels, with 2018 sales of £875,000 and 33 employees. This gradual recovery was achieved with ‘more or less the same strategy. But what we’ve done is over the intervening period we’ve just got better at what we do’.

Case C Peter Gray and PGS Ltd

Peter Gray started PGS in 1986 in partnership with his father. Peter had left school and was working for a large UK engineering company. His job was beginning to become a career when his father launched PGS. ‘Then my dad said, “I could really do with some help in the first couple of years. Can you, you know?”, so I said, “Yeah. Okay. Sounds interesting”. I’m still here’. PGS provides power generation servicing for business and institutional clients. The early years were concerned with establishing the business: ‘…we just cracked on and everything the company made, we ploughed back into the business for quite a while. We didn't pay ourselves a lot for at least probably the first 10 years’. The company grew gradually until the mid-2000s, moving to new premises and employing 35 people. Gradual growth had the firm ‘running like a sewing machine…[meaning]…people at the top had spare capacity’. It is at this point that rapid sales growth occurred, opening a second location in another part of the UK and expanding into equipment rental and sales. The business nearly doubled in size through 2011 and 2012 before contracting a little through the remainder of the decade (see Figure 1). This included refocusing on the core servicing business. At the time we interviewed Peter, had sales of £4.1 m and employed 41 people.

Case D Rebecca Maier and Industrial Dairies

Rebecca Maier and her husband joined the family dairy business in 1989, injecting £80,000 to alleviate cash flow problems following an investment in new facilities. Initial growth came a year later, with the opportunity to shift from small scale farmhouse production, selling to a distributor, to production and packaging directly for a national retailer. Establishing themselves as a reliable supplier to large retail provided the platform for further growth. As Rebecca told the story: ‘when we took it over in 1989, it was turning over £250,000, and five people. Very soon went up to eight…and very soon grew to £400,000. And then with packing, it obviously just kept going, so by the end of the ’90s, we were seven-and-a-half million, so that was just every year. Grow, grow, grow, grow, grow’. The growth through the 1990s required larger premises and the firm acquired a second production facility away from the farm. The move ‘doubled our overheads overnight, because you got two sites…and didn't double sales, so we had a few lean years’. With slower sales growth in the first half of the 2000s, and a shifting customer focus from quality to cost putting pressure on margins, they made two acquisitions to meet anticipated new demand from a large retailer. The company struggled for a number of years following the acquisition before ‘it all came together for us in 2012–13’. Steady growth followed over the next five or six years (Figure 1). At the time we interviewed Rebecca, the company had posted sales of £14m and employed 99 people.

**Case E**, James McDonald and The People People

At 18 years old, James McDonald ‘bunked out of university’, worked various jobs ‘but wanted to always have more’. After spending 18 months working as a consultant for a recruitment agency, James concluded that ‘I don't like how this industry is and I can change it’. In 1998, in partnership with his father, who had sold his own small business, and with a £30,000 overdraft facility, James launched The People People. The vision was ‘to change the way in which people recruit in the UK’. Early growth was frenetic. Turnover was over one million pounds in the first year, over two million in the second year, and £4.5 m by the end of year three; changing premises twice through this time. This early growth was driven by sales, ‘…on the phone, out and about, in the car, putting thousands of miles on my Volkswagen Polo…Going out and winning new business’. Following this initial success, performance plateaued as James’ focus waivered. However, as he reengaged and started to recruit his own staff ‘just to take some of the time from me’, the business modified its business model and enjoyed several years of steady growth, acquiring ‘some great clients’ and moving to ‘swanky offices’. The 2008 financial crisis hit the firm hard, with revenues dropping from £12m to £9m ‘pretty much overnight’. Following this, they diversified into training and ‘outplacement’ work. The next decade was marked by jumps and falls in revenue as large clients were acquired or lost. At the time we interviewed James, The People People had reported sales of £14m and employed 55 people.

Case F Stephen Bechelli and Industrial Films

Stephen Bechelli left university in the late 1970s with a degree in geography. Determined to stay close to friends and family, he found work with a large US-owned industrial textiles company in his hometown. Stephen’s father owned a small textile firm ‘making work wear, boiler suits, chef’s aprons, these types of things’. Stephen had worked during school holidays ‘earning and bit of pocket money’ and, in 1980, joined the business full-time. They expanded into industrial textiles but, through the 1980s, ‘was bumping along a lot…doing very little’. As Stephen recalled, ‘the company was not making regular profits, it was scraping along. The accounting? We knew last month’s figures six months after the year end’. For Stephen, ‘this was my future’. Shortly after Christmas 1991, Stephen convinced his father to retire and took over the company. The fortunes improved and ‘through the 1990s we began to make a bit of profit and become regular’. Growth was steady through the 2000s, with sales growth of over 20% in 2006 and 2007, taking turnover to £2.7 m. This was driven by ‘taking the opportunities and trying to make the best out of opportunities when they arrive’. These opportunities included some acquisitions and new ventures in Eastern Europe and Canada. Following these moves, sales grew by 57% in 2011. However, these expansions met with mixed success and the remainder of the decade saw some ups and downs (Figure 1). Stephen was relaxed about this period, pointing out that much of this activity was about diversification, with the business pursuing both higher and lower risk projects. As he noted, ‘if you look at the history over the last six or seven years, we have reached a reasonable, I think it’s a reasonable performance. But it never comes from the same place’. At the time we interviewed Stephen, Industrial Films had recorded sales of £5.5 m and employed 90 people.

At this point, it is worth noting that these are not young firms. Neither are they located in fast-growing, dynamic industries. When we interviewed our entrepreneurs, all the firms were over 10 years old – some much older – and all were in what we might safely call ‘traditional’ industries. Given the common observation that high growth firms tend to be younger – the issue is age, not size (Moreno and Coad, 2015; Nightingale and Coad, 2014) – and the equally common, if generally inaccurate, assumption that high growth may be found most often in technology-intensive sectors (Delmar et al., 2003)^5^, this has clear implications for how well the patterns in our data may provide the foundation for models or propositions that are suitable for general testing. That is, how well our data allow us to make ‘analytical generalisations’ (Yin, 2003). We return to these issues at the end of the paper.

As noted in the introduction, our goal was the collection of rich case data to support the development of testable research propositions that might improve our understanding of the episodic nature of firm growth. Our case data consist of archival information collected during the case firm’s participation in a university-based leadership development programme. This comprises of a variety of document types, including development plan workbooks, third party observations of board meetings, and management accounts. In large part, these data were used to establish historical growth motivations. This is complemented by performance data from 2010 to 2018 (represented in Figure 1), that records the variable sales performance of the companies following their programme participation. The larger part of the data presented in this paper comes from interviews conducted in the summer of 2019 and follow-up emails over the subsequent several months.

Interviews began with a brief description of the project or, more precisely, with a brief statement of our interest in what happens in growth companies during and after growth. From there, entrepreneurs were simply asked to tell their story. To tell us about the genesis of their venture and its evolution, questions and prompts were restricted to points of clarification and the occasional elaboration of an element of the story that seemed particularly revealing. We did not encounter the data unacquainted with the prior work on firm growth. This is not pure inductive research. Rather a reasonably strong grounding in the literature guided our search for patterns and, inevitably, shaped our interview prompts. However, in practice, we asked few questions and we are confident that the data were not contaminated by our prejudgment. The interviews were conducted by both authors, were recorded and transcribed. Each author, and a third scholar familiar with case study methodologies, independently read the interview transcripts with the goal of uncovering patterns related to growth and growing. What follows is an account of the revealed patterns in our data.

## Findings

### Growth and growth intent

The first patterns that we identify concern the relationship between past growth and growth motivations. Certainly, the notion that growth motivation (or intent or expectation) is a key antecedent to growing is firmly established in the literature (Hermans et al., 2015). However, there is some tension between the ‘implicit assumption’ that ‘motivation remains relatively stable over time’ (Delmar and Wiklund, 2008, p. 439) and ‘a received consensus in the literature that immutable intentions are unlikely’ (Dutta and Thornhill, 2008, p. 311). The former leads to conclusions concerning the reinforcing nature of success, with past successes magnifying the influence of motivation on future performance^6^. The latter, in contrast, presents growth motivations as changing over time, with this dynamic more than a simple increasing function of past performance (Achtenhagen et al., 2010).

Changing motivations were clearly evident in our cases. Tom’s observation that ‘I certainly had a time when my mojo was completely gone, and it just felt like we got three steps forward and we go four back. But I think a lot of businesses are like that’ is illustrative of this changeability. Here, our goal is to go beyond correlations between past and present motivations or performance, or studies on the influence of the *anticipated* consequences of growth on motivation (Wiklund et al., 2003) to uncover the micro-foundations of changing motivations resulting from lived experiences with growth (Wright and Stigliani, 2013).

To this end, our data suggest two clear themes: 1. Satisfaction, and 2. Growing pains. These are, respectively, the ideas that entrepreneurs are income satisficers, rather than maximisers, and that firm growth, especially early growth, is not simply hard to achieve, but is physically, emotionally and socially challenging. The first of these, the notion that there is a curvilinear relationship between income and motivation, is likely to be familiar to undergraduate economics students studying the countervailing substitution and income effects of rising wages. It also accords with longstanding evidence in the literature that income is rarely the most important variable explaining growth motivations (Wiklund et al., 2003) and that ‘the prospect of making more money is not enough to motivate further growth in most cases’ (Davidsson, 1989, p. 223). As Stephen insisted, ‘money was not the driver, and I think to me that’s an important thing. Money is not a big driver. Of course, making money is not a trivial concern’. As Peter noted, ‘Yeah. I mean, I want to make money. I enjoy the actual process of making money’. Importantly, however, making money was not seen as an end in itself. Rather, as Ged explained, ‘I’m not bothered about money particularly, but I think money is, it’s an ideal ruler. In any business, it’s looking at, it’s a measuring competition. And the further up that ruler you get with money, potentially the better you’re doing’.

The idea of money as a ‘ruler’ is consistent with an aspiration-level explanation for growth that draws on core ideas from behavioural theories of the firm (Greve, 2008). In behavioural theories, managers form aspiration levels through social comparisons with similar organisations. Faced with uncertainty, comparable firms represent relevant information about what other managers believe to be the appropriate firm size. When firm performance falls below the aspiration level, firms initiate ‘problemistic’ search for ways to improve outcomes (Cyert and March, 1963). The further below its aspiration levels a firm finds itself, the more willing it will be to take risks to improve performance. In contrast, while not actively seeking to shrink, managers of organisations operating above initial aspirations levels are less willing to take risks and will only pursue additional growth where profitability can be maintained. In this way, as Greve (2008, p. 488-9) observes, an ‘aspiration-level explanation for organisational growth is parsimonious to the point of seeming simplistic: managers seek growth when they believe that their organisation is too small’. The issue of social comparisons driving aspiration levels around income and, through this, firm size, was nicely illustrated by James reflecting on his reengagement with his business after the first growth episode: ‘And then I suppose my life started to change as well, we moved house, we moved to a better area. We started having kids that went to private school. And I actually think that had a big impact on me because I started mixing and seeing people that had a lot more than I had. And I wanted that too, and I started enjoying this lifestyle and wanting more’. The initial successes of The People People had afforded James a comfortable lifestyle but, as his society changed, his social reference points changed, and his aspiration levels adjusted upwards.

Behavioural theories of the firm tell us that managers are fundamentally satisficing individuals. Boundedly rational, they do not maximise. They satisfice. What is considered ‘satisfactory’ is a function of aspiration level, which, in turn, is set through an iterative process of social comparison (e.g. within one’s industry, with one’s peers, with one’s prior circumstances, etc.) (Gavetti et al., 2012). In a similar way, past research has pointed to entrepreneurial choices as influenced by social desirability; of the perceived status of entrepreneurs (Giannetti and Simonov, 2004). In our cases, this is exemplified by Ged’s determination to ‘show people that I’m really not just a dummy, I’m actually somebody who knows what they’re doing and is good at it’. It is also clear that decision-makers pursue multiple goals, and that these goals may trade off against each other, with the pursuit of one goal attenuating aspiration levels for other goals (Greve, 2008). Davidsson (1991) summarises the issues well. Distinguishing between perceived *Need*, *Ability*, and *Opportunity* antecedents to growth motivation he concludes that ‘Need-related issues appear more important than Ability and Opportunity (which would mean that satiation is the major reason why small firms stop growing)’ (pp. 405-6). Exhibit A presents further case data that we believe illustrate the role of satisficing in changing growth motivation (Table 1–4).

**Table 1.** Exhibit A: Satisfaction.

Case A: Ged Safety First	So, to get that kind of money anywhere else will take a lifetime of hard work within your profession to get there. So, if you’re sat at 100 grand, 75 grand, take-home pay, that’s cracking money.
Case C: Peter PGS Ltd	It’s [the business] made me a lot of money. I’ve got a nice house. I’ve got money in the bank. I’ve got nice holidays, et cetera, et cetera, and I now own the whole company, but what am I going to do with it? Am I going to flog myself now to make more money? That’s fine, but I’m asking myself, ‘What am I going to do with that money?’ Because one of the things I was frustrated and disappointed with when I look back at my father’s life for us, when he died, he was 68. He kept saying he would do these cruises and holidays and adventures soon, later. Not just yet, and they ended up not doing any of them.
Case D: Rebecca Industrial Dairies	Fundamentally, I didn’t want to change my life. I was happy with living there. I was happy to create.
Case E: James The People People	Okay, so we get to about four and a half, five million, and I’m driving quite a nice car at the time. Things are going quite well, moved to a new house. Yeah, I remember saying to people, this is just great. And so, I don’t know if it was a dip but more of a plateau.
Case F: Stephen Industrial Films	Yes, yes, what comes next? That’s the best way to put it. I think I’m happy working, I like it, but also now like my three-day weekends, and have 10 weeks holiday a year. So, I’m pretty happy, but I like being in work, and work is a very serious game and I want to play it.

**Table 2.** Exhibit B: Growing is Hard.

Case A: Ged Safety First	So then you’ve got this quite quick growth, that went from two and a half folks in an office, to now we need eight, and how to scale that up and how to manage it when you’ve never done it before. It’s really hard.
Case B: Tom Food Services	The problem with it, it’s not every small business but my observation is that you get a business to a particular size, there’s some real big questions then about where do you go now? What’s next? Stay this size, because if you stay at this size you can manage it. If I want to go beyond that, where do I get the capital? Who the hell is going to run it with me? It’s a scary place to go, and if you’ve been through liquidation like I have it’s kind of like, do I really want to do that again? That was a psychologically limiting factor. No, I do not want to go and raise a million quid or more because the industry I’m in is very capital intensive.
Case C: Peter PGS Ltd	So therefore, [growth] it’s hard work. Somebody like me who’s managing a team of people as you go through that change, it’s very, very hard work to sort of bring this team of people along with you on this journey. Because some of them, kicking and screaming, don’t want to come and some of them are all for it.
Case D: Rebecca Industrial Dairies	We literally had the village on the packing line, and at the end of 48 hours, we were quite ill, really, because if you work 48 hours non-stop, you’re quite dead. So, we were a bit wobbly, and shaky. You know, we’d been keeping feeding the staff, but yeah, it was really hard.
Case E: James The People People	I’d had nine months of serious, I only really realised it now, of serious anxiety. Of waking up in the middle of the night, hot sweats, heart racing. The only way I could get sleep was to put the discovery channel on and watch *How It’s Made*.

**Table 3.** Exhibit C: Falling Systems.

Case A: Ged Safety First	The ugly side in my eyes was the process. Didn’t like process. I’ve never liked process, and probably a part of it’s because I didn’t understand it too well. We shy away from stuff we don’t understand, don’t we? So, as the growth went on, the lack of process was highlighted.
Case B: Tom Food Services	I think it’s quite a natural reaction when you’re under cash pressure, when you’re under pressure, when it’s going wrong, you’re looking for the quick, easy fix.
Case C: Peter PGS Ltd	The bank came and they said, ‘Right’. This is like day one. All the money’s gone. Right? And then the bank said, ‘Right, we need your cash flows’. Well, I’ve never done a cash flow before. Never needed to. What’s a cashflow? So of course, start doing these cash flows and they are all projections now, things in the air, that sort of stuff. It’s like, ‘All right, okay’, but as the months and quarters rolled by, it really hit me. I need a cashflow, because I need to know where the bloody money’s coming from, and I’ve never had to have this before. All of a sudden it’s like, ‘We’ve got to get some invoices out this month, because in two months' time I need that money to pay this’.
Case D: Rebecca Industrial Dairies	You keep going back and you actually find holistically you’ve got at root cause, you’ve not planned, your staff aren’t trained, you’ve got some fundamentals wrong with your systems and procedures and structure that are actually causing all these knock-on effects which are manifesting themselves in bad luck.
Case E: James The People People	I remember saying to Gerry, why the hell are we keeping all this data, us recording all this data? And he’d say, I don’t really know yet, but we will know why one day. He said because if you haven’t got it, you can’t interrogate it, you can’t look at it.

**Table 4.** Exhibit D: Hiring Challenges.

Case A: Ged Safety First	I did try and bring somebody in who was more senior and more knowledgeable. And he wasn’t right for small business. So, I used the recession to get rid of him.
Case B: Tom Food Services	I employed a guy for two years and he drove me mad, he was really not good enough to do the job. But he came from [a large retailer], but he taught me one thing and I’ll be eternally grateful to him. I just was obsessed with wastage, gross margin, and I had completely lost sight of if you’re not in the van you can’t sell it. So he made me stop and he said, ‘you do realise you’re actually killing yourself because you’re trying to control the wastage so much we have nothing to sell’.
Case C: Peter PGS Ltd	We have tried some high calibre people. We’ve brought some high calibre people into the business, so-called, lots of experience, earn a lot of money, promise a lot of deliverables, and when they get here, they can’t do it.
Case D: Rebecca Industrial Dairies	I’m not saying she wouldn’t have got us out of those problems. She would have, I’m pretty sure. She was an absolute whiz, but I wasn’t good enough to work alongside her, and represent...It wasn’t a good team. Could have been. We had compatible skillsets, but it wasn’t a good team, really. I don’t know why. I believe it was my lack of ability to appreciate her skills at the time.
Case E: James The People People	But I got rid of Gerry because of a huge cultural change within the business. He was running the business very differently than I would. And he went away on a skiing holiday, and all the senior team, of which there were four or five senior managers, four out of the five of them, male and female, were in tears saying that they can’t continue doing what they’re doing at the moment for Gerry.
Case F: Stephen Industrial Films	What happened in the Canada was I hired somebody who actually a friend to run the business, and I put a friend in charge of the business who had run a business and had been quite successful. I hadn’t really fully understood why he was successful, and what was going on, and how get got into the circumstances, because everybody doesn’t always tell you the whole truth. I realized after a while it wasn’t going to work because he couldn’t do the right things. He didn’t understand the business well enough, and I couldn’t control it well enough, because where they were...You know, it’s five-hour time difference, it’s a long flight, not easy to get to. We did a lot of good work, they made profit one year, but then lost a lot of money.

The second factor that appears to bear upon changing growth motivations is how difficult the experience of growing was. When researchers note that ‘growth is hard’, they typically mean that ‘growth is hard to achieve’, reflecting on the rarity of growth (Nightingale and Coad, 2016). However, as Penrose (1959) insisted, growth is not simply about a change in size. Rather, growth, as a process of internal development, is accompanied by a variety of managerial challenges. These are explicit in the organisational lifecycle literature (Lewis and Churchill, 1983; Hambrick and Crozier, 1985; Greiner, 1989), where they are presented as problems that must be overcome to enable the firm to transition to the next stage in the lifecycle. We discuss some of the organisational and strategic responses to these challenges in the next section. Here, however, our interest is in the extent to which these challenges alter motivations (Garnsey and Heffernan, 2005).

Simply put, ‘growth creates problems’ (Garnsey et al., 2006, p. 13). Ged captures this well: ‘What you’ve got to be careful of is growth creates lots of change. Growth only means that our job gets harder, more difficult, more complex, whatever, so why...How do they buy into all of that?’. Growth brings about two kinds of challenges: The first is ‘an atmosphere of frenzy’ (Hambrick and Crozier, 1985, p. 35) that subjects decision-makers to the kinds of time compression diseconomies identified by Dierickx and Cool (1989) and discussed elsewhere in the entrepreneurship literature (Steffens et al., 2009; Koryak et al., 2018). The second is that the firm is suddenly bigger, ‘without any aptitude or preparation for being big’ (Hambrick and Crozier, 1985, p. 35). Intriguingly, the literature suggests that entrepreneurs often anticipate the negative effects of some of these challenges. For instance, past work reflecting on the influence of entrepreneur *expectations* of growth challenges on growth motivation, notes that ‘fear of reduced control and employee-wellbeing stand out as the most powerful growth deterrents’ (Davidsson, 1989, p. 219; see also Wiklund et al., 2003) and may help explain the rarity of ‘continued entrepreneurship’ (Davidsson, 1991). In our case data, growth’s impact on employee well-being is powerfully illustrated by Peter’s observation:

‘I think growth is exciting and I think it’s great for the people at the top who are driving growth. What you’ve got to be careful of is it creates lots of change. And a lot of people don’t like it. When you’re doing this growth, you’re on a curve and you’ve been thinking about it for such a long time and planning it, and when it’s being executed, you’re right on the front of the curve. All these other people, they’re way back here, they don’t know what’s coming. They sort of, you’re hitting them with a tidal wave of change and they just...You need to prepare for that. You’ve got to sort of get them ready for it. Some people just aren’t ready for it or don’t want it or don’t see what they get out of it’.

When the excitement fades for the ‘people at the top’, ‘new procedures are experienced as constraints [and] motivation and commitment decline’ (Garnsey and Heffernan, 2005, p. 687). Fast-growing companies are under considerable strain as social organisations (Hambrick and Crozier, 1985). New employees are hired who are unfamiliar with each other and with the firm. The ‘tidal wave of change’ identified by Peter affects morale and staff burnout and turnover may be high as ‘people came and went and we had all sorts of challenges’ (Peter). The number of decisions that must be made, and the information required for decision making, grows rapidly. Entrepreneurs find themselves wearing many hats (Mathias and Williams, 2018) and the quality of decision-making declines (Hambrick and Crozier, 1985). As Stephen reflected, ‘I was doing all that, if I wasn't doing that nobody else in here has a network of external contacts like I do…it’s getting more difficult and if I leave things, things don't get done’. For our entrepreneurs, ‘there was an incredible amount of hard, physical effort went into getting this right. And then perfecting the model is overstating it but trying to keep the model together and working so that it could deliver growth and profit’ (Ged). As Tom concluded ‘It’s a scary place to go, and…it’s kind of like, do I really want to do that again?’. Exhibit B (Table 2) provides additional quotes from our interview data that exemplify how difficult the experience of growing was for our entrepreneurs.

Taken together, income satisfaction and vivid memories of the challenges associated with rapid growth had a powerful effect on desires for future growth. This is not to suggest that the entrepreneurs became anti-growth. Rather, our entrepreneurs still ‘enjoy growing the business’ (Peter). But, having experienced both rapid growth and poorer performance, they are now focused on ‘trying to build something which is sustainable’ (Ged). Recognising that what may be good for growth, may not be good for profitability (Nason and Wiklund, 2015), our entrepreneurs began to reflect more carefully on the reasons for growing. Peter captures this well: ‘Everybody says growth is a wonderful thing, but I’m not here for growth. I’m here for profit, and that’s not the same. I’m looking for profitability. If growth doesn't give me that, then I don't see the point in doing it’. While early growth was ‘exciting’, it was also sobering.

**Figure 2.**
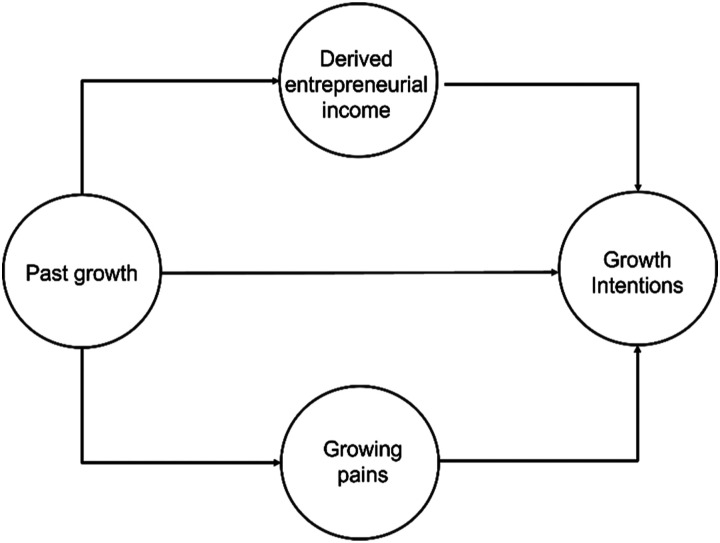
The mediating roles of income and growth experiences.

Figure 2 summarises our observations on the relationship between realised income and the difficulties experienced in achieving growth, on one hand, and subsequent growth intentions, on the other. Where past growth increases entrepreneurial incomes beyond initial aspirations, it will serve to alter the entrepreneur’s attitude towards risk-taking and to reduce further growth ambitions. Similarly, where the entrepreneur recalls growing as ‘hard’, these recollections will, in turn, dampen their enthusiasm for further growth. Of course, past work has noted relationships between income (Cassar, 2006) and identified barriers to growth (Lee, 2014; Douglas, 2013), respectively, and growth intent. However, our interest is in these only insofar as they are consequences of past growth that bear on the likelihood of growing again. To this end, one might think of them as analogous to the behavioural desirability and perceived behavioural control elements of Ajzen’s (1991) theory of planned behaviour (TPB). TPB is, itself, a common framing device for studies of growth intention in (entrepreneurial) small firms (Hermans et al., 2015). However, as with the study of growth generally, the relationship is linear rather than recursive. Yet, past growth clearly influences income and associates with variable experiences of the growing process. These, in turn, are likely to substantially mediate past growth’s relationship with growth intent. Certainly, they may not be the only influences on changing aspirations and intentions. Factors such as changing health or family circumstances may also bear on entrepreneurial ambition. However, changing income and recollections of hardship are the direct consequences revealed by our data and we believe that they are likely to represent common patterns that may contribute, powerfully, to explanations of the rarity of growth persistence.

### Changing skills and strategies nevertheless

While income and experience had made our entrepreneurs more cautious about growth, they remained ambitious. However, the extent to which ambition is translated into business development is affected both by the complexity of growth and by the entrepreneur’s ‘degree of volitional control’ (Delmar and Wiklund, 2008, p. 439). That is, by their ability to identify opportunities and to structure their organisations and develop suitable strategies. In this regard, several patterns were apparent in our data.

The first of these patterns concerned the centrality of the entrepreneur to the initial high growth episode. This is a frequent observation, with the size and simplicity of the firm allowing the entrepreneur to take centre stage (Mueller et al., 2012). However, while the entrepreneur is often characterised as ‘wearing many hats’ (Mathias and Williams, 2018), it was the entrepreneur’s ‘discovery abilities’ (Steffens et al., 2009) that were evident in our cases. As Stephen recalls ‘a lot of it’s putting yourself about to get opportunities and then taking the opportunities when they arise…That’s it, it’s taking the opportunities and trying to make the best out of opportunities when they arrive…So, I was doing all that’. Initial growth was sales driven, with the entrepreneur driving that process. It was James ‘…on the phone, out and about, in the car…Going out and winning new business’. It was Ged ‘playing to my strengths, which were talking to people’. It was Rebecca’s belief that ‘that’s what a company needs. It needs sales’.

However, as sales grew, weaknesses in systems were revealed. Ged offers a particularly egregious example: ‘we found one day, by accident, there was an excess of £50,000 not billed out…We got paid and everything. But, to this day, I know we will not have billed everything, we won't have found it all because we just weren't set up right’. As Peter observed, ‘all this growth needed extra resources to actually process all the paperwork and to pack and ship all the goods that were coming in and going out of business. The margins were just evaporating with all the extra overheads that we were needing’. The inadequacy of existing systems and the challenges of introducing new systems while growing, is a familiar theme in the growth lifecycle literature (Lewis and Churchill, 1983; Greiner, 1989). Exhibit C (Table 3) provides further examples of the systems challenges faced by our case firms as they experienced growth.

The experiences of our entrepreneurs are consistent with the notion of a ‘curse of fast growth’ (Zhou and Van der Zwan, 2019); with rapid growth leading to a variety of ‘internal challenges and difficulties that reduce or eliminate the benefits of growth’ (Senderovitz et al., 2016, p. 394). Small firms that quickly become bigger ‘must modify their organisational arrangements’ (Hambrick and Crozier, 1985, p. 37) and develop formal systems in areas of planning and control, and in recruitment and compensation for their expanding workforce. However, in all our cases, a shifting focus towards systems appeared relatively straightforward. Our entrepreneurs were able to recognise the importance of replacing ‘first-hand direct’ activities with managerial ones (Mueller et al., 2012, p. 999). As Ged noted, ‘you’re getting processes and systems in place better. You’re making sure that Ged Martin doesn't run through the middle of the business. So, you’re trying to remove yourself whilst making sure that growth still happens’. Volery et al. (2015, p. 117) observe that since ‘entrepreneurs are doers…unsurprisingly exploitation appears to be the default activity for all of them’. This may overstate the case. But a changing emphasis from discovery to exploitation – from sales to systems, from entrepreneurship to management – presaged changing firm performance in our cases. Mathias and Williams (2018, p. 262) contend that, as firms grow, entrepreneurs must wear fewer hats; they must make decisions about ‘which roles to give up, which roles to retain, and which new roles to adopt’. In our cases, this was, at heart, a decision about the entrepreneur’s relative emphases on discovery – or exploration – and exploitation (Steffens et al., 2009).

The notion, and importance, of ambidexterity is entrenched in the strategy literature. This is the idea that a firm must ‘engage in sufficient exploitation to ensure its current viability and, at the same time, to devote enough energy to exploration to ensure its future viability’ (Levinthal and March, 1993, p. 105). Steffens et al. (2009) suggest that an emphasis on discovery alone may allow the firm to generate short-lived growth that is difficult to sustain. This may be manifest in more variable performance, with bursts of high growth followed by periods of poor performance in the absence of an effective exploitation capability. In contrast, a focus on exploitation is likely to lead to more stable performance and profitability but is unlikely to result in sustained high growth. Ambidexterity, then, is key to persistent growth. However, behaving ambidextrously requires entrepreneurs to ‘manage contradictions and competing goals, engage in paradoxical thinking and fulfill multiple roles’ (Raisch et al., 2009, p. 687). Unsurprisingly, entrepreneurs appear more likely to emphasise *either* exploitation or exploration (Volery et al., 2015), deciding to devote more and less time to these two competing activities.

In our cases, Peter, Rebecca, Tom and Stephen embraced a systems and efficiency focus as their business grew. Ged and James continued to play to their strengths in product development and sales. All, implicitly, recognised the importance of ambidexterity or, at least, recognised the changing skills that their growing companies required. For instance, Peter reflected that ‘I realised when we’re at that point that the team that had got me from there to there was not a team that could do it again, take me from there to there. They didn't have the skills to do that’. In a similar vein, Ged recalled that ‘I didn't recruit properly because my initial recruitment was to help me to get to 150 grand turnover. Okay? But very quickly, and I can’t remember exactly, we got the numbers up to £650,000 turnover. So, the people I’d recruited at that level weren't really capable of getting me sustainably to this level and being good, but I didn't sack them and look for more, because you stick with folks. I was managing folks that weren't right for the job and I was trying to make them fit’. The development of complementary managerial skills as firms grow is central to Penrose’s (1959) theorising. Simply put, growing firms need to hire and develop a management cadre that offers complementary capabilities to support and expand the scale and scope of a firm’s operations (Coad et al., 2014a). The need to hire new, complementary management resource was recognised by all our entrepreneurs. The urgency to wear fewer hats was nicely captured by James’ recollection that ‘I wanted people that I could grow, I could develop…We were doing four and a half million, we were growing. I couldn't do anymore, didn't have the time to do it’.

As our entrepreneurs devoted more time to exploitation or recognised the increasing need for better exploitation, they set out to hire explorers or exploiters, respectively. Four of our entrepreneurs reduced their focus on entrepreneurial behaviours – on opportunity seeking – to focus on managerial tasks, such as improving systems and processes, prioritising profitability over sales. These individuals tried to hire people with entrepreneurial skills to fill the gaps their changing attentions had left behind. Two of our entrepreneurs continued to focus on opportunities. In these cases, the goal was to hire people with managerial skills, to ensure that systems and processes kept pace with growing sales. Regardless of whether they were trying to hire entrepreneurs or managers, explorers or exploiters, our entrepreneurs experienced mixed success. James’ reflections on a former senior hire are illustrative:

‘Gerry was very well respected, very capable individual. And was much better than I was at making things happen, almost in a way. I was still good at the ideas, but Gerry did things. The data that he created was fantastic, the metrics that we had in our business, to measure our business, which we’ve still got now…So made Gerry MD, which he did for at least two or three years, I think. And the business continued to grow all the time during Gerry’s tenure. In fact, when I got rid of Gerry, he was absolutely shocked because it was just at the time when things were going quite well’ (see also Exhibit D).

These missteps in hiring appear to be driven by two factors. The first of these was the reluctance of our entrepreneurs to fully give up former roles. Where an entrepreneur’s former role becomes a role identity ‘this can create friction between who entrepreneurs are and who their ventures need them to be’ (Mathias and Williams, 2018, p. 264). Rebecca acknowledged this challenge:

‘The technical manager couldn’t be a technical director because I was there as a technical director, so he didn’t have any power. I didn’t know how to empower him. The marketing man couldn’t have his own say because, again, I was there saying “No, you can’t say £19.32. It’s £19.69.”…And I was going that direction, this other man was going that direction. And he wasn’t empowered, so he wasn’t successful’.

Another pattern in our cases was the adhocracy of hiring, especially for senior hires. What little research that exists around hiring patterns in growth firms suggests that successful firms ‘dedicate extraordinary attention’ to recruiting and developing managers (Hambrick and Crozier, 1985, p. 40). More recent work points to the ‘profound effects’ of staffing and human resource management on a growing firm’s performance (Senderovitz et al., 2016, p. 398). Yet, in our cases, hiring processes for key individuals were often informal and unplanned. When discussing a senior hire, Ged explained that ‘I tripped over somebody again’. This was echoed in Tom’s description of a senior employee as ‘a fabulous guy’ and the hiring process as ‘again, that was just serendipity’. Key hires often came from close social or business networks, with issues of trustworthiness and loyalty looming larger than formal assessments of competence. Of course, these informal processes also led to good hiring outcomes. However, bad hiring outcomes appeared more common, leading our entrepreneurs to a preference for, as Stephen explained, ‘developing talent, not hiring people who are experienced’. Inevitably this has implications for growth. On the one hand, Penrose (1959) pointed to the absence of external markets for managers with internal knowledge and experience and positioned internally developed expertise as critical to growth. On the other hand, rapid growth puts a strain on internal management development as prospective managers struggle to train and acclimatise new employees and become distracted from operational concerns (Coad et al., 2019). Moreover, expertise that is developed wholly internally is unlikely to lead to a management team of ‘individuals with extensive human capital and industry experience but with diverging mental models’ (Coad et al., 2014b, p. 297), that past works has suggested high growth firms should ‘strive’ for. Top Management Team (TMT) heterogeneity is frequently positioned as critical to widening the ‘attentional set of the organisation’ and enhancing ambidexterity (Koryak et al., 2018, p. 415) (Table 4).

Given this evidence, it would be tempting to echo the lament that static perspectives on human capital have dominated the growth literature (Demir et al., 2017). However, we prefer to emphasise that a dynamic perspective rests on understanding changing human resource requirements as a consequence of growth. Before the firm grows, systems and processes are relatively simple, and entrepreneurs may comfortably wear many hats. Tom captures the stereotype of the entrepreneur as generalist well:

‘A SME owner knows about all sorts of stuff because they’ve just got to. They may not be an expert in it, but my God most SME owners can probably tell a lawyer something about employment law that they don’t know if they’re not an employment lawyer because they don’t have the niche. They’ve got this wide, huge, and they’ve got to be really creative in their thinking and they’ve got to learn themselves because nobody else is going to do it for them’.

However, as the firm grows it becomes more complex and specialist skills are required. It is growth that triggers the consideration of ‘which hats to keep wearing, which to remove and which new hats to adopt’ (Mathias and Williams, 2018, p. 263). The decisions made at this point, and the processes enacted to support those decisions, bear heavily on the subsequent performance of the firm. In concert with changing motivations, the unwillingness or inability of our entrepreneurs to resolve the ambidexterity conundrum explained their inability to sustain or repeat rapid growth.

**Figure 3.**
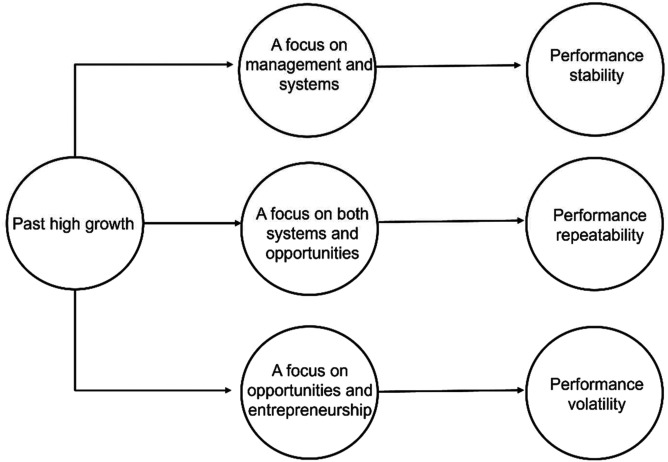
The mediating role of managerial focus.

As before, we attempt to summarise our observations in Figure 3. Growth firms inevitably face challenges around the adequacy of initial resources for sustained growth and performance (Garnsey et al., 2006). Of particular concern is the extent to which systems are able to meet the demands that growing sales place on upstream value chain activities (Hambrick and Crozier, 1985). Our intuition (supported by our data) is that most ambitious entrepreneurs will recognise the need to resolve these challenges and, in the medium to long term, to find ways to maintain efficiency and to simultaneously explore new opportunities to develop their venture. Their capacity to do the latter will largely rest on two things: the willingness to ‘give up hats’ and the ability to hire and empower the right people (Mathias and Williams, 2018). Recognising that few entrepreneurs (perhaps none) will be able to continue to wear many hats in an increasingly complex organisation, the path that the organisation takes is likely to tend towards one of three, with these mediating the relationship between past and future performance. While we are not convinced that ‘the biggest burden a growing company faces is having a full-blooded entrepreneur as its owner’^7^ it does seem likely that sustaining growth requires that the entrepreneur find ways ‘to delegate responsibility and detach themselves…and be happy with it’ (Davidsson, 1989, p. 223). Of course, other things may influence the stability, repeatability and volatility of performance after initial growth (including and, perhaps, especially external factors). However, we believe that over a long enough window it will be the ability to develop competent human resource practices that support ambidexterity that will distinguish those firms that are able to repeat or sustain growth from those who experience a single episode of growth or more punctuated performance.

## Discussion

Our point of departure was an increasingly familiar frustration with the literature on high growth firms and its influence on policy (cf. Shane, 2009; Mason and Brown, 2011, 2013). As Moreno and Coad (2015, p. 220) observe ‘findings of low persistence should be ringing alarm bells among policy makers and researchers’ (p. 220). It is clear, not only that sustained growth is incredibly rare but, equally, that many more firms than are commonly thought enjoy a period of high growth. In this light, Hart et al.’s (2021, p. 17) contention that ‘there’s no such thing as a High Growth Firm…only firms that have high-growth episodes’ is compelling.

Following this, we believe that the episodic nature of firm growth calls for greater attention to growth consequences, to counterbalance the legion of studies of growth causes; the latter invariably characterised by low explanatory and predictive power (Wright et al., 2015). In this, we echo Eshima and Anderson (2017, p. 777) that ‘a fruitful approach to building an integrative model of firm growth is to consider growth’s proximal consequences in smaller, more manageable studies’. In part, we hope that this will help stimulate discussions about ‘how much’ growth firms ought to pursue (Demir et al., 2017). While studies occasionally observe that growth ‘is not always good news for a firm’ (Steffens et al., 2009, p. 126), a normative perspective prevails. Beyond this, our cases strongly suggest that it may be growing rapidly that lays the foundations for not growing rapidly again, at least in some firms, or for some entrepreneurs.

Here the distinction between firms and entrepreneurs is not insignificant. Our richer data is largely drawn directly from our entrepreneurs. It is their changing perspectives, their recollections and their decisions that are the consequences of their experiences of rapid growth. It is these that bear on motivations and actions following growth. In this, we respond to Wright and Stigliani’s (2013, p. 4) ‘call for a shift in emphasis beyond the firm to include the entrepreneur level. Such a shift is particularly important, since entrepreneurial firms do not make decisions about growth – entrepreneurs do’.

More specifically, our goal was to develop propositions about growth consequences that might help shed light on why sustained growth was quite so rare. We wanted to be able to ask better questions of the sophisticated large-scale datasets that are increasingly available to researchers. Our cases do not permit statistical generalisations, but we believe that the varied experiences of our entrepreneurs reveal common patterns that might form the basis of analytical generalisations. And that these, in turn, may be practically framed as hypotheses.

To this end, the patterns regarding growth motivations appear easiest to interpret. The growth motivations of entrepreneurs are moderated by increasing income and wealth. We anticipate that the relationship between income (or wealth) and growth motivation will exhibit initially increasing returns, followed by diminishing returns. The point of inflection on this curve is likely to be a function of aspiration levels. Aspiration levels will be set through an iterative process of social referencing and by entrepreneurial opportunity costs (Cassar, 2007). They may be adjusted upward (as in the case of James), however motivations that rest on aspiration levels are not likely to be amenable to manipulation through simple policy interventions; although a longer term focus on local entrepreneurial culture and an emphasis on role-modelling may help raise entrepreneurial aspiration levels across the board (Capelleras et al., 2019).

We also propose that growth motivations will be moderated by the extent of ‘growing pains’. The more difficult the initial experience of high growth – physically, socially, emotionally – the less likely entrepreneurs are to seek to repeat it. Of course, changing motivations resulting from growing pains may be more tractable. Where these ‘pains’ are related to systems failures or human resources, as was most common in our cases, it ought not to be beyond the capabilities of the various ‘policy’ actors to devise interventions that better prepare entrepreneurs for these challenges.

Certainly, income (or wealth) and growing pains are not the only factors that may affect attitudes towards growth. In our cases, changing family circumstances (e.g. older spouses, dependent children, family ill health, and so on) were frequently discussed in relation to commitment to the business. In many ways, this recalled early social development perspectives on entrepreneurship (Gibb and Ritchie, 1982) and more recent work that explores the relationship between entrepreneur age and relative attachment to social or economic value goals (Brieger et al., 2020). While these influences were not directly consequent on past growth experience, they serve to reinforce the multiplicity of entrepreneurial motivations and should further caution researchers against presumptions of growth motivations as primary.

Beyond changing motivations, our cases illustrated the changing role of human resources in driving initial growth and constraining future development. Prior work has suggested that high growth firms are more likely to hire ‘marginal’^8^ employees during their initial growth, but attract older individuals, already in employment, in later stages of growth episodes (Coad et al., 2014b). Human resource dynamics are also reflected in Brown et al.'s (2017, p. 436) observation, hidden away in a footnote, that ‘a recent survey of HGFs found that 74% of HGFs ranked access to talent as one of their top three growth constraints…this would suggest that a key growth bottleneck for HGFs is effective recruitment and talent management’. The dynamic nature of human resource challenges was strongly evident in our cases. As growing increased complexity, widening the managerial attention set required (Koryak et al., 2018), our entrepreneurs attempted to hire complementary skills. The implicit goal was ambidexterity. Those who had begun to focus on exploitation tasks tried to hire explorers. Those who continued to focus on exploration and discovery tried to hire exploiters. Inadequate hiring processes and an unwillingness to relinquish control resulted in failure more often than success. In consequence, our exploitation-focused entrepreneurs enjoyed steady, if unspectacular performance, while our exploration-focused entrepreneurs experienced more variable performance, punctuating bursts of high performance with periods of poor performance. Following this, we propose that, in the absence of considered human resource planning and a willingness to delegate, ‘good’ firms will be unlikely to build capable and ambidextrous Top Management Teams and, as a result, unlikely to sustain or repeat high growth.

Of course, our intention is not to suggest that ambidexterity, fostered by considered human resource management, is the only strategic influence on firm growth. Or, indeed, on sustained or repeated growth. Much as the general literature on the causes of firm growth has identified numerous influences, of inconsistent impact, we anticipate that researchers will be able to identify a similar cafeteria of factors that encourage or constrain second or continued growth (see, for example, Parker et al. (2010) on the importance of dynamic management strategies). However, our interest was more specific. We were concerned with how experience of past growth influenced choices and strategies for further growth and development. To this end, the dominant theme in our case study data concerned the recruitment and retention of management talent to meet changing business needs, and the implications of choices made here on the firm’s ability to simultaneously exploit existing capabilities and explore new opportunities. The challenges that our firms faced were a mixture of ‘Penrose effects’, associated with the integration of new employees into the society of a growing small firm (Lee, 2014), and the entrepreneur’s reluctance to relinquish functions that had become an important part of their role identity (Mathias and Williams, 2018). Here again, it may be possible to design interventions that ameliorate these challenges. When we reflect on the curricula of our *new venture creation* or *strategic entrepreneurship* courses (and their policy equivalents), it is remarkable how little attention is given to human resources and human resource management. Rather, these courses continue to be dominated by discussions of innovation, opportunity recognition, venture capital, and the like.

A final, supplementary, implication flowing from our data is that ‘good’ firms are much more commonplace than the literature on high growth firms or gazelles would have us believe. Our cases are good firms, providing good jobs. That they have not continued their early rapid growth does not diminish their ‘goodness’ and is not inevitably a result of bad decisions or the ‘cynical and unfair view that holds that the early managers are inherently unsuited to the demands of a larger firm’ (Hambrick and Crozier, 1985, p. 37). Levels of satisfaction (Davidsson, 1991) meant that the entrepreneurs were more cautious risk takers, as anticipated by an aspiration-level theory of firm growth (Greve, 2008). They continued to pursue opportunities, but on their terms and with improved profits rather than sales or employment as the goal. Given the ‘the extraordinary force of mortality’ in the small business sector (Anyadike-Danes and Hart, 2018, p. 46), their ability to survive and, by and large, thrive is as much evidence of ‘continued entrepreneurship’ as consecutive periods of high growth (Davidsson, 1991)^9^.

## Limitations and future research

Our exploratory study has some obvious limitations. It rests on a small number of cases, purposively sampled. Our cases are older (both the entrepreneurs and their firms) and in traditional sectors. They are also located in the north of England, with all that entails^10^. It may be that different patterns will be evident among younger entrepreneurs, leading younger firms, competing in more dynamic knowledge- and technology-intensive sectors, surrounded by other technology entrepreneurs. This is an open empirical question. It is, of course, not our intention to suggest that the patterns we observe can be easily generalised to the population of smaller firms. While our intuition is that many of these patterns will hold or be shaped in fairly predictable ways by things like initial motivation (e.g. growing to sell is likely to be experienced differently from growing to keep), our goal was to nudge growth conversations towards a consideration of consequences and to help us ask better questions.

The limitations in the current study suggest avenues for future research. Most directly, we hope to encourage testing of the propositions developed here in larger and more diverse samples. For instance, most empirical research linking incomes to growth intentions has either been concerned with pre-entrepreneurial incomes and opportunity costs (e.g. Cassar, 2006) or with how the anticipated income effects of imagined growth bear on motivations to grow (Davidsson, 1989; Wiklund et al., 2003). We believe that there is considerable scope for investigating the proposition that, at some level of relative income or wealth (i.e. some aspiration level), entrepreneurs will demonstrate a preference for minimising losses over maximising gains. Understanding whether this holds generally and, if so, how this aspiration level is set is likely to be revealing. One obvious extension concerns potential differences across institutional contexts. For instance, intriguing recent work has suggested that institutional arrangements that encourage the flourishing of billionaires differ markedly from those that encourage self-employment (Sanandaji and Leeson, 2013). This, and similar work exploring institutional influences on the extent of ambitious entrepreneurship (Bowen and De Clercq, 2008), has typically concerned itself with formal or regulatory institutions, to the extent that these are measurable. However, if aspirations are socially referential it seems likely that normative and cognitive institutions will be critical in determining aspiration levels and in shaping how income and wealth dynamically figure in the configuration of the multiple goals that entrepreneurs pursue over time.

Beyond this, we do not consider that our propositions are exhaustive. Rather, we hope to encourage further work that acknowledges the episodic nature of growth and recognises that, in addition to causes, episodes have consequences that are likely to be critical to understanding the non-linearity of growth paths. In this vein, we anticipate that two lines of research may be particularly promising. The first concerns the multiplicity of motivations that entrepreneurs pursue, how these interact, and how they are altered by prior performance. As discussed earlier, past work has viewed growth as an ‘acquired taste’, with past positive outcomes reinforcing growth motivations, and vice versa (Delmar and Wiklund, 2008). However, this narrow perspective perpetuates the myth of business growth as ‘the very essence of entrepreneurship’ (Pierce and Aguinis, 2013, p. 321). Instead, it seems likely that many firms exhaust growth in larger part because growth motivations are superseded by other motivations. As in our cases, entrepreneurs may continue to have ambitions for their firms, but these ambitions will be tempered by other considerations.

A second line of research concerns the contingent role of past performance on future business development. Although ‘bouncing back’ may be typical for firms that have experienced high growth episodes, there are a small number of firms who are able to repeat their good performance (Capasso et al., 2014). In much the same way as experiences of rapidly growing made it less likely that our entrepreneurs would grow rapidly again, understanding these cases of persistence is likely to involve understanding how past growth experiences shape the circumstances that lead to further growth and development. We view this as mirroring Coad and Rao’s (2008) observations on the variable influence of innovation on growth across the growth distribution. In an important contribution, these authors observed that the effect of innovativeness on growth was strongest for those firms above the 90^th^ quantile of the growth distribution and had a negative effect on firm growth in the lowest quantiles. The implication of this work is that what influences performance is likely to vary across the performance distribution. As an extension, we believe that the factors driving growth will also vary across the *past* performance distribution. It seems reasonable to speculate that growing out of decline, or from a period of stability, will require different sets of capabilities and activities than those required to sustain or repeat high growth.

## Conclusions

While our specific goal was to develop testable propositions that would help us in our own research on firm growth, our more general goal was to encourage consideration of growth consequences, at least as a complement to the large volume of work on growth causes. Specifically, our data suggest that entrepreneurial motivations and behaviours are altered by past performance and are likely, in their turn, to bear on future performance.

**Figure 4.**
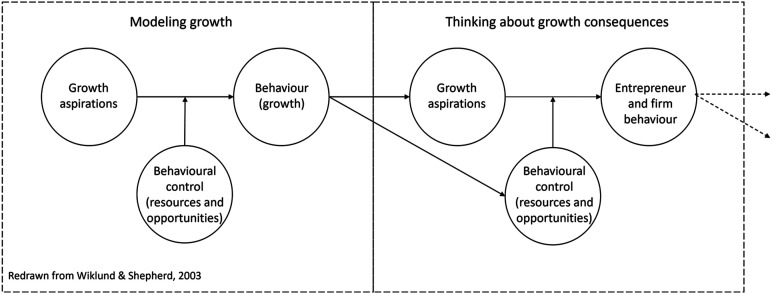
Growth consequences and growth paths.

We illustrate this in Figure 4, where the left-hand pane reproduces a typical approach to the study of growth (Wiklund and Shepherd, 2003, p. 1923). Following the Theory of Planned Behaviour (Ajzen, 1991), it models the relationship between aspirations and realised growth as moderated by behavioural control, where behavioural control is represented by bundles of resources and strategies and by opportunities. The right-hand frame illustrates how a focus on consequences might contribute, with both aspirations, resources and perceptions of opportunities altered by past performance and, in their turn, influencing subsequent behaviour, and so on as the path unfolds.

Recognising that growth has proximate consequences that bear directly on future performance positions growth processes as path dependent (Zhou and Van der Zwan, 2019). Specifically, a focus on consequences is concerned with the processes and mechanisms that are manifest in the arrows linking past behaviour and aspirations and past behaviour and resources and opportunities, and, subsequently, the arrows linking these to future behaviour. The recursive nature of the relationship between the causes and consequences of firm growth underlines the importance of studying growth paths, rather than growth events (Garnsey et al., 2006; Miozzo and DiVito, 2016) and places the study of growth paths centre stage in the developing literature on firm growth (Brenner and Schimke, 2015). A paths approach would allow us to better balance causes and consequences and, we believe, build richer explanations.

## Notes

1 Here, growth ‘consequences’ are those things that result directly from the process of growing. Of course, some of these may also be ‘causes’ of further growth (e.g. changes in the quantity and quality of resources). However, in the recursive relationship between causes and consequences, we believe that a clearer focus on consequences might shed considerable light on why high growth is rarely repeated.

2 As part of a larger project on growth consequences funded by the Social Sciences and Humanities Research Council of Canada, econometric work is currently underway using survey and administrative data in the UK and Canada. Through this, we test some of the ideas raised in this paper.

3 We have changed the names of the individuals and the firms and, where sensible, relocated them in place and sector.

4 Unless otherwise indicated, quotes are taken directly from the transcripts.

5 Delmar et al.’s (2003) ‘Super Absolute Growers’ are found disproportionately in ‘knowledge intensive manufacturing industries’ although, taken as a whole, the paper offers a much more nuanced perspective on firm growth.

6 Delmar and Wiklund (2008, p. 449), for instance, find evidence for the ‘temporal stability of growth motivation’ in bivariate correlations between past and current motivation of 0.27 and 0.39 for employment and sales motivations, respectively.

7 Here Kets de Vries is actually quoting an Entrepreneur, Derek du Toit.

8 ‘…young individuals, poorly educated workers, single household members, immigrants, individuals with prior unemployment, and individuals without self-employment experience are more likely to be employed by an HGF’ (p. 295).

9 Here there may be parallels with the recent literature on ‘degrowth’ (e.g. Kallis, 2011), which emphasises the potential for economies to thrive and prosper in the absence of continued economic growth. We are grateful to a reviewer for identifying this link, which we believe suggests intriguing lines of research in different development settings.

10 As fellow Northerners, we particularly enjoyed Peter’s observation that ‘Yeah. I’m saying, well, again because I’m a northerner, when I pop me clogs, I want there to be no money left. I want to have spent it all’.
